# COL6A1 loss of function mutation underlie atrioventricular septal defects in down syndrome patients

**DOI:** 10.1186/1755-8166-7-S1-P24

**Published:** 2014-01-21

**Authors:** Priyanka Ghosh, Pranami Bhaumik, Subrata K Dey

**Affiliations:** 1 West Bengal University of Technology, Department of Biotechnology & Biological Sciences, Kolkata, West Bengal, Pin-700064, India

## Background

The present investigation was undertaken to explore the role of COL6A1 variants on cardiovascular septal defects in Down syndrome.

## Materials and methods

We sequenced the entire reading frame of COL6A1 in the samples from Kolkata and adjoining areas. Four hundred participants were included in the genetic association study and they were stratified as Down syndrome (DS) with atrioventricular septal defect (AVSD), DS without AVSD, euploid with AVSD and euploid without AVSD. DNA of Down syndrome individual with atrioventricular septal defect and Down syndrome individuals without AVSD were sequenced for the SNP analysis of COL6A1 gene.

## Results

A missense mutation p.E887K in exon 35 of COL6A1 gene was identified in three DS patients with complete AVSD and was absent in control population. The causative potential of a sequence variation was evaluated by bioinformatics software like Mutationtaster, MutPred and SOPMA which predicted. The mutation was predicted to be deleterious and is predicted to affect normal protein function. Genetic analysis of the mutation carrier's available family members showed that the substitution co-segregated with AVSD transmitted in an autosomal dominant pattern. The p.E887K variation was automatically predicted to be disease causing.

## Conclusions

Our data suggest that missense mutation p.E887K in exon 35 of COL6A1 mutation may serve as a potential biomarker for diagnosis of AVSD in individuals thus suggesting potential implication for early prophylaxis and personalised treatment of AVSD.

